# Adjuvant Ipilimumab in High-Risk Uveal Melanoma

**DOI:** 10.3390/cancers11020152

**Published:** 2019-01-29

**Authors:** Eric Fountain, Roland L. Bassett, Suzanne Cain, Liberty Posada, Dan S. Gombos, Patrick Hwu, Agop Bedikian, Sapna P. Patel

**Affiliations:** 1 Department of Hematology/Oncology, The University of Texas MD Anderson Cancer Center, Houston, TX 77030, USA; efountain@mdanderson.org; 2 Department of Biostatistics, The University of Texas MD Anderson Cancer Center, Houston, TX 77030, USA; rlbasset@mdanderson.org; 3 Department of Melanoma Medical Oncology, The University of Texas MD Anderson Cancer Center, Houston, TX 77030, USA; scain@mdanderson.org (S.C.); lposada@mdanderson.org (L.P.); phwu@mdanderson.org (P.H.); abedikia@gmail.com (A.B.); 4 Department of Head and Neck Surgery, Section of Ophthalmology, The University of Texas MD Anderson Cancer Center, Houston, TX 77030, USA; dgombos@mdanderson.org

**Keywords:** adjuvant, ipilimumab, high-risk, Class 2, uveal melanoma, clinical trial

## Abstract

Uveal melanoma is a common intraocular malignant tumor that is uniformly fatal once metastatic. No effective adjuvant therapy currently exists to reduce the risk of distant metastasis after definitive treatment of the primary lesion. Immunotherapy has been used effectively in the adjuvant setting in locally advanced cutaneous melanoma. We performed a Phase I/II clinical trial of adjuvant ipilimumab in high-risk primary uveal melanoma with distant metastasis-free survival (DMFS) as the primary objective. A total of 10 patients with genomically high-risk disease were treated: three at a dose of 3 mg/kg and seven at 10 mg/kg. Two of the seven patients at the higher dose had to discontinue therapy secondary to grade 3 toxicity. At 36 months follow-up, 80% of patients had no evidence of distant disease (95% CI, 58.7–100). With recent advancements in CTLA-4 inhibition, PD-1 inhibition, and combined checkpoint blockade, immunotherapy is a promising avenue of treatment in uveal melanoma. Further clinical trials are needed to elucidate the role of immunotherapy in the adjuvant setting.

## 1. Introduction

Uveal melanoma is the most common primary intraocular malignant tumor in adults and represents approximately 5% of all melanoma diagnoses [1,2]. Primary uveal melanoma is definitively treated with brachytherapy, proton beam radiation, or enucleation [3,4], and clinical evidence of metastatic disease at presentation is rare [5]. Despite definitive treatment, approximately 50% of patients with uveal melanoma will relapse with a uniformly fatal metastatic disease and have a median overall survival period of less than 12 months [6,7,8,9]. Features of the primary tumor prognostic for an increased risk of distant metastatic disease include tumor size, AJCC staging, and genomic analysis demonstrating monosomy 3 or DecisionDx-UM high-risk molecular gene signature [10,11,12,13,14]. Currently, there is no consensus regarding adjuvant therapy for reducing the risk of distant metastases. Attempts to use dacarbazine, low-dose interferon, hepatic arterial fotemustine, and sunitinib have either failed to show an overall survival benefit or been limited by study design [15,16,17,18].

The role of immunotherapy in uveal melanoma is currently unclear. The use of checkpoint inhibitors in the metastatic setting has shown no conclusive benefits to date [19,20,21,22,23,24,25,26,27], and no studies have been published regarding the use of immunotherapy in the adjuvant setting. In cutaneous melanoma, the use of adjuvant CTLA-4 inhibitors and PD-1 inhibitors has been proven efficacious for locally advanced disease [28,29,30]. Here, we report the results of a Phase I/II trial of adjuvant ipilimumab in high-risk patients with uveal melanoma after definitive treatment of the primary tumor.

## 2. Materials and Methods

Between February 2013 and September 2013, we enrolled 10 patients in a single-institution, open-label, single-arm phase I/II clinical trial of adjuvant ipilimumab for high-risk uveal melanoma. The primary endpoint of the study was distant metastasis-free survival (DMFS) at 36 months. The study was conducted in a dose-finding fashion followed by dose escalation. Dose-finding proceeded in a 3 + 3 fashion at a starting dose level of 3 mg/kg with a second dose level of 10 mg/kg. Administration of ipilimumab either at 3 mg/kg or 10 mg/kg occurred intravenously over 90 minutes. In the induction period, ipilimumab was administered every 21 days for a total of 4 doses. Beginning at week 24, ipilimumab was administered every 12 weeks for 3 doses to complete treatment at week 48. Treatment was discontinued for death, disease progression, unmanageable toxicity, or withdrawal of consent. The protocol was approved by the institutional review board at the University of Texas MD Anderson Cancer Center (protocol code: CA184-187) and conducted under the principles of the International Council of Harmonization and Good Clinical Practice. Drug and funding for this investigator sponsored research study was provided by Bristol–Myers Squibb (New York, NY, USA) to support the study and was registered with www.clinicaltrials.gov as NCT01585194, IND 106207. All patients provided written informed consent prior to enrollment. The sponsor had no role in data collection, analysis, or interpretation, or in writing of this report.

### 2.1. Patient Selection

We enrolled patients age ≥18 years with a history of uveal melanoma definitively treated with enucleation or irradiation within 12 months of enrollment. Patients were required to have an Eastern Cooperative Oncology Group (ECOG) performance status of 0 or 1 and no evidence of metastatic disease on CT chest, abdomen, and pelvis with oral and intravenous contrast. Patients defined as being at high risk of recurrence based upon the presence of (1) Class 2 high-risk molecular gene signature using DecisionDx-UM^®^ (Cohort 1) or (2) monosomy 3 or apical thickness on baseline echography >8 mm (Cohort 2) were eligible for inclusion. Additional pertinent inclusion criteria were as follows: The ability to give informed consent, at least 42 days from prior immune therapy; absence of active or chronic infection with HIV, Hepatitis B, or Hepatitis C. Pertinent exclusion criteria included: The presence of a second primary malignancy from which the patient has been disease free for less than 2 years; autoimmune disease; prior treatment with ipilimumab or another CTLA-4 inhibitor; and chronic use of systemic corticosteroids. All patients in the study were enrolled in cohort 1 on the basis of a Class 2 gene expression profile.

### 2.2. Patient Monitoring for Adverse Effects and Metastasis

Dose-limiting toxicities (DLTs) were graded according to the National Cancer Institute Common Terminology Criteria for Adverse Events (CTCAE); Version 4.0; likely, possibly, or probably related to treatment. Patients were monitored with complete blood counts with a differential, comprehensive metabolic panel, and thyroid function tests at the induction treatment visits, at weeks 12, 16, 20, and 24 after the induction phase, and every 12 weeks during the maintenance phase. The absence of metastatic disease was monitored by CT body imaging at baseline, week 24, and every 6 months thereafter until removal from study. After completion of maintenance therapy, patients who continued to be followed at MD Anderson continued to receive CT imaging every 6 months thereafter, while those who were followed locally were monitored at physician discretion and were contacted periodically for survival follow-up.

### 2.3. Statistical Analysis

The primary objective of this study was DMFS at 36 months from the time of study enrollment. DMFS was estimated using the method of Kaplan–Meier and was calculated from the start of treatment until distant disease progression. Patients were censored at their last known date of contact. Target DMFS was 70% at 36 months, with a null hypothesis of 50% at 36 months seen with the current standard of care of observation and no adjuvant therapy. The planned enrollment to detect this difference was 38 patients in cohort 1 over a 2-year enrollment period, giving this cohort 90% power with a type I error of 0.05. Data analysis was performed using GraphPad Prism 7 Software (La Jolla, CA, USA).

## 3. Results

### 3.1. Patient Demographics

Planned enrollment in cohort 1 (Class 2) and cohort 2 (Monosomy 3 or apical height >8.0 mm) of the study was 89 patients. A total of 10 patients were enrolled and treated, all in cohort 1, after which the study was amended due to changing priorities to investigate nivolumab in combination with ipilimumab in the metastatic setting. Consequently, no patients were enrolled in cohort 2. Of the 10 patients, median age at diagnosis of primary tumor was 58, and 60% of the cohort were male. All 10 patients carried the high-risk DecisionDx-UM^®^ Class 2 gene expression profile. For definitive control of the primary tumor, 2 patients underwent enucleation, 1 had proton beam therapy, and the remaining 7 were treated with brachytherapy. The average number of days between definitive therapy and initiation of adjuvant ipilimumab was 142 days. Regarding prior local therapy, 1 patient received thermotherapy after brachytherapy, and 2 received intraocular bevacizumab. A third patient received intraocular bevacizumab prior to definitive therapy as a consequence of misdiagnosis of the primary tumor as macular degeneration (Table 1). 

### 3.2. Treatment

The median number of doses of ipilimumab was 6. Four patients received all 4 induction doses, and 8 received at least 1 dose of maintenance therapy. In patients where an induction dose was skipped due to adverse events(s), resolution or treatment of toxicity allowed treatment to resume. Two patients discontinued treatment after 2 doses due to toxicity, both of which were treatment-related, while no patients discontinued treatment for disease progression. Following a 3 + 3 dose escalation, the first 3 patients received induction and maintenance ipilimumab at 3 mg/kg with no DLTs. Therefore, the subsequent 3 + 3 patients received ipilimumab at a dose of 10 mg/kg with no DLTs during the DLT period. Therefore, the last patient received ipilimumab at 10 mg/kg in dose expansion. 

### 3.3. Efficacy

**Figure 1:**
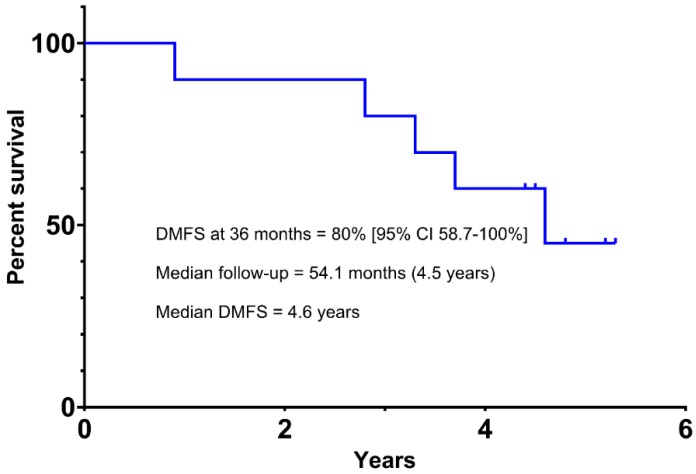
Distant Metastasis-Free Survival (DMFS) in High-Risk Uveal Melanoma Patients Receiving Adjuvant Ipilimumab.

**Figure 2:**
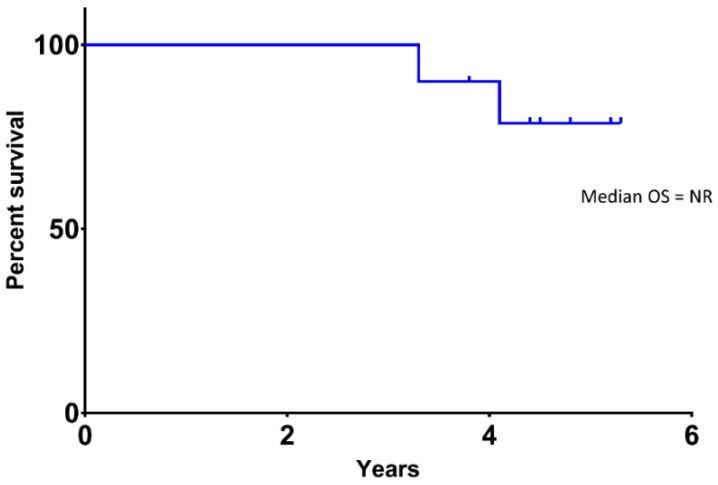
Overall Survival (OS) in High-Risk Uveal Melanoma Patients Receiving Adjuvant Ipilimumab.

The median follow-up period was 54.1 months (4.5 years). The primary endpoint of distant metastasis-free survival (DMFS) at 36 months was estimated at 80% (95% CI, 58.7–100) using the method of Kaplan–Meier (Figure 1). At the time of data cutoff, 4 patients had developed distant metastatic disease with a median time to progression of 3.25 years, and there was one death unrelated to melanoma or treatment. Median DMFS for the total study population was 4.6 years. Overall survival (OS) is shown in Figure 2, and median OS was not reached for the total enrolled population. For the patients who developed metastatic disease, median OS was 20.8 months (1.7 years). One patient died from metastatic disease and another died from causes unrelated to melanoma. The remaining 8 study patients remain alive, with 3 patients on active treatment for metastatic disease. 

### 3.4. Adverse Events

Two (20%) of the 10 patients treated at 10 mg/kg tolerated only 2 cycles of induction ipilimumab before removal from study secondary to grade 3 vasculitis and colitis/transaminitis, respectively. All three patients treated at the 3 mg/kg dose completed at least 5 of 7 cycles; grade 3 rash and grade 3 endocrinopathy were the most severe toxicities in the two patients who failed to complete all 7 cycles, respectively (Table 1). Nine of 10 patients had at least one grade 3 toxicity; one patient had grade 4 toxicity (i.e., temporal arteritis), resulting in persistent blindness despite prolonged steroid taper. The most common organ systems affected by treatment were the skin (e.g., rash or pruritus) in all 10 patients and liver function abnormalities in 9 of 10. Two patients developed adrenal insufficiency with persistent need for maintenance steroids at last follow-up. A complete list of toxicities by system and grade is presented in Table 2. 

## 4. Discussion

To our knowledge, this is the first prospective study of adjuvant ipilimumab in primary uveal melanoma. In our cohort of high-risk patients, DMFS at 36 months was 80%. All 10 patients were risk-stratified as DecisionDx-UM^®^ Class 2, which carries a historical DMFS of 50% at 32–36 months [11,31]. Grade 3/4 toxicity requiring discontinuation of therapy during induction phase was seen in 2 of 10 patients.

We acknowledge that the small sample size of 10 patients and comparison to historical controls are limitations of this study. Prospective studies in uveal melanoma tend to have lower accrual, given the rarity of disease, and use of historical control is not uncommon. While this data is promising, it is not possible to infer from our analysis that adjuvant ipilimumab conferred a statistically significant DMFS benefit. Recent literature has also suggested that the largest basal diameter (LBD) of the primary tumor carries prognostic significance independent of Class 2 gene expression profile and may represent a potential confounder [32,33]. While the sample size of our study limits additional subset analysis, 8 of 10 patients had an LBD of at least 12 mm, which confers the highest risk of distant metastases [32,33]. 

Additional confounders that may have resulted in a higher than expected DMFS include selection bias of potentially younger, healthier patients, and early termination of the study. The former likely contributed to the prolonged overall survival of 20.8 months for those who developed metastatic disease compared to historical controls [6], as these patients were subsequently treated with local therapy for liver metastases in addition to enrollment in several metastatic uveal Phase I/II clinical trials. The latter resulted in variability in monitoring for distant metastasis, as those followed at MD Anderson continued to receive serial CT imaging, while monitoring locally was at the discretion of the local physician. At the time of data cutoff, the study team was able to confirm all alive, disease-free patients would continue to undergo surveillance for metastatic disease. 

Data from studies of immunotherapy in cutaneous melanoma can provide useful information regarding the path forward in uveal melanoma. Ipilimumab is a CTLA-4 inhibitor first shown to have significant efficacy in metastatic cutaneous melanoma [34,35]. With the advent of PD-1 inhibitors, PD-1 monotherapy has been shown to have superior progression-free survival compared to ipilimumab, along with an improved toxicity profile, in metastatic disease [36]. 

In the adjuvant setting, high dose ipilimumab at 10 mg/kg was administered to patients with resectable stage III cutaneous melanoma in the EORTC 18071 trial, which demonstrated improved progression-free survival with median 26.1 months versus 17.1 months compared to placebo, though notably 43% suffered immune related adverse effects [28,29]. A recent publication comparing adjuvant nivolumab to ipilimumab in resectable stage IIIB, IIIC, and IV resected cutaneous melanoma demonstrated a 12-month recurrence free survival of 70.5% to 60.8% in favor of the PD-1 inhibitor. Grade 3 or 4 toxicities were present in only 14% of the nivolumab group but seen in 45.9% of the ipilimumab group [30]. 

In light of this data, it is not surprising that recent studies in uveal melanoma have focused on the therapeutic effect of PD-1 inhibitors given their greater efficacy and more acceptable toxicity profile compared to CTLA-4 inhibitors in cutaneous melanoma. However, in a publication of approximately 50 evaluable patients with metastatic uveal melanoma treated with anti-PD-1 monotherapy, the objective response rate (ORR) was 3.6% [24]. Therefore, the role of PD-1 inhibition in the adjuvant setting for uveal melanoma remains unclear. As a caveat, however, CTLA-4 inhibitors as single-agent therapy in recent phase 2 studies of metastatic uveal melanoma have shown an equally unimpressive 0% ORR, with significant grade 3–4 toxicities in approximately 35% of cases [27,37]. Given these results, it is unclear why the promising data presented here may suggest a potential role for CTLA-4 inhibition in the adjuvant setting, but further validation in subsequent studies will be required to establish a definitive clinical benefit. To potentiate the expected toxicity profile of single-agent CTLA-4 inhibitors, especially at higher doses as seen in this study and adjuvant trials in cutaneous melanoma [28], future avenues of research are expected to focus on the role of combined checkpoint inhibition with both PD-1 inhibitors and CTLA-4 inhibitors. These combinations have shown a synergistic effect on outcome in metastatic disease [38,39], and the utility of this combination in the adjuvant setting has been studied in cutaneous melanoma (CheckMate-915, NCT03068455) and is being explored in high-risk uveal melanoma patients as well (NCT03528408).

## 5. Conclusions

There remains a great need for effective adjuvant therapy in high-risk primary uveal melanoma given the significant mortality of metastatic disease. Here, we present the first pilot trial of immune checkpoint blockade in the adjuvant setting for uveal melanoma, with 80% (95% CI, 58.7–100) of patients disease-free at 3 years compared to 50% for historical controls. Further clinical trials are needed to establish the role of CTLA-4 and PD-1 inhibition for the adjuvant treatment of high-risk uveal melanoma.

## Figures and Tables

**Table 1:** Baseline demographics for adjuvant ipilimumab patients.

Patient ID	Age	Gender	Definitive Treatment Modality	Additional Local Therapy Prior to Enrollment	Size (mm)	T Stage *****	DecisionDx UM Class	Days Elapsed ***	Number of Cycles of Ipilimumab	Ipilimumab Dose (mg/kg)	Baseline LDH (ULN 618 IU/L)	Reason for Discontinuation
1	35	M	Brachytherapy	Yes, thermotherapy	16 × 15 × 8	3	2	204	7	3	386	Completed Therapy
2	45	M	Brachytherapy	-	12 × 10 × 3	1	2	63	5	3	421	4th induction dose and last maintenance dose held due to Grade 3 transaminitis and Grade 2 fatigue and hypothyroidism
3	58	F	Brachytherapy *	Yes, avastin	15 × 12 × 4	2	2	77	6	3	523	3rd induction dose held due to Grade 3 rash
4	74	F	Brachytherapy	-	13 × 13 × 4.6	2	2	161	7	10	552	Completed therapy
5	60	M	Proton beam therapy	-	21 × 17 × 10.1	4	2	111	2	10	402	Only 2 doses given due to Grade 4 vasculitis
6	59	F	Brachytherapy	Yes, avastin	13 × 10 × 4.4	2	2	317	2	10	428	Only 2 doses given due to Grade 3 diarrhea, Transaminitis
7	65	M	Enucleation	-	14 × 12 × 11	3	2	83	7	10	357	Completed therapy
8	50	F	Brachytherapy	-	6 × 5	N/A	2	179	6	10	287	3rd induction dose held secondary to Grade 3 hypophysitis
9	57	M	Brachytherapy	**	8 × 8.1 × 2.7	1	2	67	5	10	417	4th induction dose held secondary to Grade 2 hypophysitis; last maintenance dose held due to Grade 3 transaminitis
10	58	M	Enucleation	-	15 × 14 × 9	3	2	153	7	10	592 ****	Completed therapy

* In combination with cryotherapy and radiation; ** received intraocular avastin prior to diagnosis of uveal melanoma; *** from definitive therapy to initiation of ipilimumab; **** LDH obtained after initiation of therapy; ***** all tumors located within the choroid and/or ciliary body.

**Table 2:** Adverse events.

Adverse Events	Grade 1–2 (n/10)	Grade 3–4 (n/10)
Constitutional	7	0
Endocrine	4	1
Gastrointestinal	5	2
Hematologic	0	0
Hepatic	6	3
Infectious	1	0
Musculoskeletal	1	0
Neurological	2	0
Pain	0	0
Psychiatric	0	0
Pulmonary	1	0
Renal	0	2
Skin	8	2
Vascular	0	1
